# It’s not all about control: challenging mainstream framing of eating disorders

**DOI:** 10.1186/s40337-023-00752-9

**Published:** 2023-02-19

**Authors:** Dawn Branley-Bell, Catherine V. Talbot, James Downs, Carolina Figueras, Jessica Green, Beth McGilley, Claire Murphy-Morgan

**Affiliations:** 1 Department of Psychology, PaCT Lab, Northumbria University, Northumberland Building, City Campus, Newcastle Upon Tyne, NE1 8ST UK; 2 Bournemouth University, Poole, UK; 3 Patient Representative, Royal College of Psychiatrists, London, UK; 4 Universitat de Barcelona, Barcelona, Spain; 5 Leeds and York NHS Partnership Foundation Trust, Leeds, UK; 6 Adjunct Faculty, University of Kansas School of Medicine-Wichita, Wichita, USA; 7 Department of Psychology, Northumbria University, Newcastle Upon Tyne, UK

**Keywords:** Eating disorders, Control, Healthcare, Stigma, Anorexia nervosa, Bulimia, Discourse

## Abstract

**Background:**

The concept of control has long been suggested as a central factor in eating disorder (ED) aetiology. The concept is now so mainstream that it risks being used in a potentially reductionist, stigmatising or otherwise harmful manner. In this paper, we explore and discuss our positions on the use of control-related terminology for EDs.

**Methods:**

The authors of this auto-ethnographic position paper include academic researchers, individuals with lived experience and clinicians (not mutually exclusive). In sharing our experiences and observations, we aim to raise awareness of the wider impacts that control framing can have on ED perceptions, treatment, recovery and individuals’ lived experience.

**Results:**

We argue that although control can play a role in some ED experiences, an overemphasis upon this factor to the exclusion of other conceptualisations is not beneficial.

**Conclusions:**

To mitigate against pathologisation of an individual, it is important to challenge a discourse that can lead to EDs being perceived as something ‘wrong’ with the individual, rather than a consequence of life events or other environmental influences. We identify priorities for the future for researchers, clinicians, policy makers and the wider public.

## Plain English summary

Control has often been described as a central factor within Eating Disorders (EDs). Whilst control can play a role in ED experiences, we argue that overemphasis upon this factor can result in other important factors being overlooked. For many individuals, EDs are the consequence of life events and/or other environmental influences. With this in mind, discourse which overemphasises control (e.g., rather than *coping*) can inaccurately portray EDs as something ‘wrong’ with the individual. It is important to challenge this discourse to encourage more appropriate perceptions of EDs. In turn, this could improve understanding and treatment of EDs, reduce stigma, and promote recovery.

## Background

It is estimated that there are over 55.5 million people worldwide with eating disorders (EDs) [1], with approximately 1.25 million in the UK [2]. For many years, EDs have frequently been described using a control framing, for instance being associated with a ‘need for control’ over uncertain and potentially negative aspects of life [3, 4]. This has dominated discourse during the COVID-19 pandemic, with researchers reporting that people with EDs turned to disordered behaviours to compensate for a perceived loss of control [5, 6]. People with EDs across several studies [7–9] have discussed the role of control, and the meanings they assign to their experiences should not be discounted. However, in this paper we question whether control should be the *primary* narrative—or whether excessive emphasis and focus upon this particular factor can be detrimental.

Negative terminology is often used in relation to EDs, both in general day-to-day lives and in treatment settings. Researchers have long critiqued conceptions of the “devious”, “difficult”, and “dishonest” person with ED(s) [10–16]. Yet, discussion of these issues in relation to control are notably absent. In society, describing someone as ‘controlling’ is often a negative descriptor linked to undesirable qualities and behaviours such as manipulation. Likewise, describing someone as ‘needing to be in control’ is often linked to stigma around weakness or neuroticism. These descriptors are often thought of as stable traits of an individual’s personality, i.e., resistant to change and not dependent upon circumstance or context. Such framing locates the problem *within* the individual, rather than being more critical of the wider social circumstances a person might find themselves in. Stopping at control and ignoring what the person is seeking to have control *of* (i.e., the context) leads to thin descriptions which risk pathologising the individual [17].

From our own experiences as researchers, clinicians and individuals with lived experience, we have become aware of the prevalence of control framing; It has become so commonplace that often it is used without any conscious reflection on its appropriateness. Whilst control plays a role in EDs, we argue that care must be taken to critically consider how and when it is appropriate to talk about control (over other factors). Reference to control must be sensitive to the individual and non-promoting of negative stereotypes or perceptions; the latter is particularly important as EDs are known to be one of the most stigmatised disorders [14, 16].

**Fig. 1:**
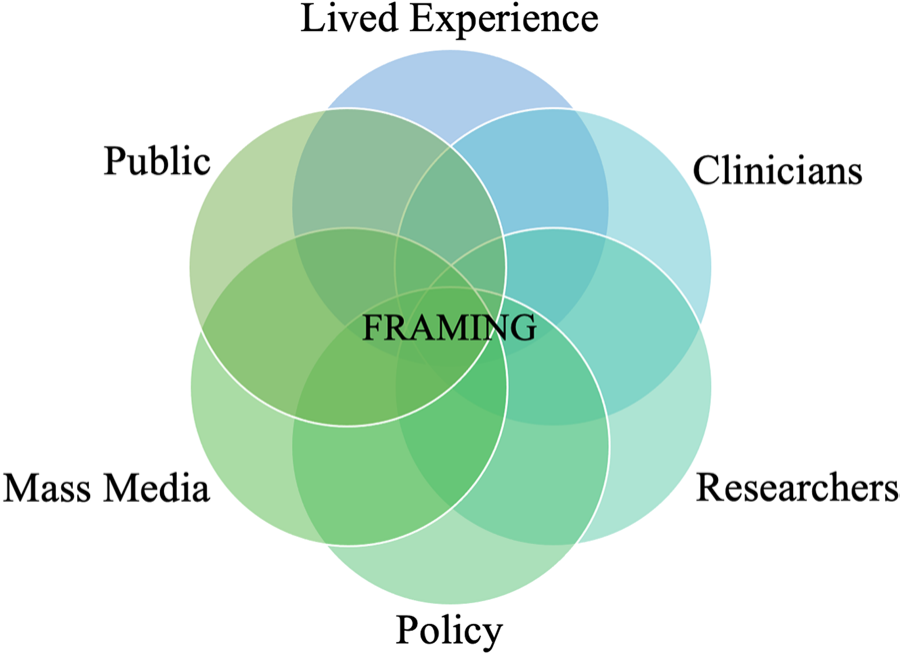
Mapping the influence of control framing around eating disorders

Through our collective experiences we reflect on how control framing can negatively impact an individual’s well-being and ED recovery progress, in addition to broader aspects of their lives. Overemphasis upon control can foster stigma of individuals with EDs; can reduce hope for recovery through attributing EDs to a fundamental ‘issue’ with the individual rather than a reaction to their experiences and/or environment; and can result in a reductionist approach to care which overlooks other significant factors. In addition to impacting individuals’ experiences, clinical approaches and public attitudes—control framing can also impact how EDs are perceived within the media, policy and research (see Fig. 1).

Existing research suggests that many individuals regard control, to some degree, as *one* of many aspects of their ED. Control also features in many approaches to treatment, e.g., the various forms of Cognitive Behavioural Therapy [CBT], and Dialectical Behaviour Therapy [DBT]. We emphasise that we are not disputing that control is a potential factor, we are arguing against the excessive use of control as the central framing when talking about EDs – and its use in a simplifying, reductionist manner. Murray et al. [18] highlight that individuals with EDs who do not display body shape and weight concerns (sometimes referred to as “non-fat phobic”) are described by the transdiagnostic cognitive–behavioral conceptualisation of EDs (CBT-E) as having a core psychopathology which is “a need for control, in general”. Interestingly, in their study [18], there was no evidence that a need for control is a central factor for this group. It is possible that inconsistent results around control as a central factor may be attributed to an easily lost, but important, distinction—CBT-E was introduced by Fairburn [19], who described control as one of the main *maintaining* factors, i.e., that which underlies continuation of the ED. This is an important distinction as this differentiates from a need for control being a *precursor* to, or *causal* factor of, an ED. This is also more in keeping with the recognition that ED behaviors initially serve a purpose as a coping mechanism and response to negative life events. Unfortunately, it is easy for subtle or nuanced distinctions to be lost in common language. For example, take Radically-Open Dialectical Behaviour Therapy (RO DBT) which seeks to teach individuals to be “more open, flexible” and less focused on “excessively self-controlling” [20]. Whilst some of the techniques used within this approach may be helpful (e.g. identifying healthier coping mechanisms), RO DBT is also officially described as a therapy for “disorders of overcontrol” [21, 22]—again, this use of language places excessive emphasis upon control.

We believe it is imperative to stop and think about the impact of an overemphasis upon control, not only for individuals with EDs—but also for their friends, family, loved ones, clinicians, and researchers. In doing so, we hope to encourage a move away from reductionism and towards language that is more reflective of the many complex factors underlying EDs.

## Methodology

This position paper uses an auto-ethnographic approach, whereby the authors have drawn on their range of perspectives as individuals with lived experience, clinicians and/or researchers (quotes are shown within the sub-group which they apply to—e.g., quotes in the clinician section are only from authors with clinical experience and similarly for the lived experience and researcher groups). It is important to note that our perspectives are not mutually exclusive, with some of us occupying intersecting perspectives.

In writing and collaborating on this paper the authors individually reflected upon and critiqued their experiences of control framing and associated impacts. After initial individual reflection, the authors discussed their opinions and experiences and formulated the central narrative and aim of this paper—that is, to construct a narrative of their intersecting positions on the control discourse around EDs.

## Perspectives on control

In this section we share our thoughts and reflections from three main perspectives: individuals with lived experience; academic researchers working within this field; and clinicians working specifically within ED healthcare services.

### Individuals with lived experience

As three individuals with lived experience of EDs, we reflected on our experiences and how ‘control’ has been discussed in our healthcare journey. This is not to say our experiences are representative—everyone’s experience is unique, and they may highlight different factors in their own journey.

One of the first things we reflected upon was whether notions of control were introduced to us by our clinicians. Two of us felt that this had happened and shared concerns over the appropriateness and helpfulness of placing emphasis upon control. For the third individual in our group, the lack of this experience was associated with receiving treatment in the late 1970s.

*Within the context of my lived experience, the notion of “control” has always been a foreign concept. In fact, at the peak of my anorexia, and before being diagnosed, I recall the first time someone advised me to “control other things rather than my weight”. At the time, I was clueless about what that person meant. What kind of control was she talking about?*

Upon reflection, encountering discussions around control during ED therapy and treatment is perhaps not surprising given that control is so dominant in today’s conceptualisation of EDs [3, 4, 21]. However, this language may subsequently frame and shape how an individual perceives and experiences their ED. For all three of us, potential problems arise when there is an *overemphasis* upon control as a central driving factor universally underpinning all EDs. From our perspective, we also feel that the relevance of a control framing may vary depending upon the type of ED. Attempting to apply an overall framework could lead to confusion and could also lead to other significant factors being overlooked. For us, our EDs have not been all about control, they have been about *coping*, for example coping with negative past experiences or current negative feelings such as feeling overlooked, lost, isolated, trapped or unsafe.

*Whilst my experience of eating disorders may represent a way of mediating my experience of the world, I don’t think this is about control at heart. Control has been, at times, a mechanism - a means to an end. The end hasn’t been “to be in control”. If anything, that end has been safety. I wasn’t ever attempting to achieve control, or mastery of my body, thoughts and feelings. I’ve only ever wanted to be safe.*

*In hindsight, and after years of recovery, I would say that my eating behaviors were instrumental, at least in the beginning, to cope with fear of the unknown and the dread of uncertainty. Anorexia brought me predictability, and a methodical daily routine.*

There are other reasons why an emphasis upon control could be unhelpful, not least that it suggests there is something fundamentally ‘wrong’ with the individual; rather than recognising that, for many people, EDs represent a coping mechanism to deal with negative environmental experiences. This framework can result in people feeling stuck and hopeless about recovery due to feeling that their ED is a fundamental part of who they are as a person; something which is “*unmovable, intractable, untreatable*”.

*Whilst control can be a vehicle for meeting other needs, the professionals who have treated me have consistently seized upon the mechanism of control as “the thing”. This has left me feeling like the underlying drivers of my distress have remained unseen.*

*My lived experiences of EDs have included both anorexia and bulimia since my mid-teens. Now in my 30s, I live with a “severe and enduring eating disorder”, a label which comes with connotations of something unmovable, intractable, untreatable.*

We would urge healthcare professionals to start thinking outside of the narrative of control. The least we can do is ask whether the focus on control a good fit for the individual patient. We need to be more open to different ways of making sense of EDs, beyond the needs which any “controlling” behaviours might be serving.

## Researchers

As three researchers within this field, we have been prone to adopting control framing and have written papers ourselves where this is apparent. For example, we recently published work on the role of control during the pandemic, reporting that ED behaviours served as an ‘auxiliary control mechanism’ [8]. It was only through our recent work in this area, and our many fascinating conversations with experts by experience, that we started to critically evaluate this discourse.

We are particularly interested in how language around mental health conditions can have a significant impact on how individuals are viewed by others, and/or how they view themselves. For instance, we recently wrote about the negative impact of fat-shaming language during the UK Government’s ‘Better Health’ campaign; and how a potentially well-intended campaign had the potential for harm [23]. We also know that people can internalise language and public beliefs/attitudes around mental health disorders, thereby perpetuating self-stigmatisation and influencing how they make sense of their individual experience [24, 25].

We have identified preliminary evidence that other researchers have also begun to question whether EDs are ‘all about control’. For example, a 2017 study [18] found control was not a key maintaining mechanism of restrictive eating behaviour within their participant group. We would like to take this a step further and raise awareness around the need to critique current frameworks across the field.

It is important to emphasise that we are not denying that aspects of control can, and often do, play a role in EDs. Indeed, for some individuals control may play a key role, and this must be respected, explored and unpacked. Individuals should never be advised against using language which they feel accurately portrays their own experiences. This relates to work by Sampson [26] which highlights the need for psychology to not restrain individuals voices by enforcing a dominant dialogue, but rather that individuals must be given the opportunity to use their own voice. For example, clinicians and researchers should work to help individuals unpack and reposition themselves in relation to discourse.

*We are by no means discounting the vast body of work that has investigated the role of control, but instead urge others to critique this concept and recognise it as one of the factors in a complex network.*

Our aim is not to remove any mention of control but to encourage critical reflection around whether control is the most appropriate *central, overarching framing* when discussing EDs [3, 4, 21]. There are many different factors, and differences, involved in any one individual’s experience of an ED. By placing excessive emphasis on control, the concern is that we may overlook some of these factors; focusing on a framing associated with negative stigma and perceptions, and in doing so simplify individuals lived experience in a manner which could be detrimental. Similarly, this framing can influence public and social perceptions of EDs, we wish to encourage a move away from negative perceptions and stereotypes of an individual concerned with control and promote more supportive perceptions of an individual using a coping mechanism to deal with something in their lives (past or present).

*Small differences in language can have a big impact. Imagine two people being described to you, one is described as needing control, the other is described as using a coping mechanism. Which would you have more empathy for? This is just a small, quick example of how language can affect our preconceptions.*

Control framing appears to have originated primarily within the context of Anorexia Nervosa (AN) [27] but has been adopted more widely and is now often applied to EDs more generally [28]. This tendency is reflective of the portrayal and understanding of EDs in our society at the moment.

*Despite not being the most prevalent ED, AN continues to be the most widely recognised and publicly portrayed. To the extent that often the terms ‘Anorexia’ and ‘eating disorder’ are used interchangeably by the mass media and the general public.*

Research suggests that there are distinct differences between EDs [29]. Therefore, we have significant concerns over the adoption of a universal framing around control, which could be inaccurate and misleading. We call for researchers (such as us) and others within the field to start more consciously critiquing the framing they use; and to ensure individuals have ample opportunity to unpack, challenge and/or reposition the language used when talking about, or describing, their ED experiences. We would also like to see improved policy guidance around appropriate and responsible media reporting when publishing about EDs (e.g., akin to media guidelines for reporting on suicide [30]).

### Clinicians

As two service providers, one within England’s National Health Service (NHS) and one and one in private practice in the US (also recovered from an ED for nearly 40 years)—we reflected on what role the control framework can play in relation to treatment and support.

In our experience, the notion of control can be raised in early conversations by the service user, *not* the service provider. There are several possible reasons why this may be the case. Firstly, this could illustrate that the notion of control does play a valid role for some individuals. Secondly, this could be a consequence of adopting social norms (and the dominant discourse) around EDs. Thirdly, the term could have been introduced from another healthcare source prior to the individual arriving for treatment with us. Of course, there are countless speculative reasons, and the answer is likely to be a combination of many of them. For instance, it is likely that control does play a role in some EDs but that this may become overemphasised and/or simplified through common language. This does not take away from individuals who feel that control is a meaningful concept for them, in these instances this language should be validated and respected.

*My first contact with service users typically includes a period of assessment and psychological formulation (the collaborative effort between therapist and service-user to summarise their individual difficulties and make sense of why they developed and what is keeping them going), and within these early conversations the idea of control is frequently raised. Not by me or other members of the multi-disciplinary team- but by the individual suffering. Therefore, for many individuals with an eating disorder the concept of control is meaningful, and validating their lived experience should be our first priority.*

Once conversations progress with our service users, they often reveal negative past experiences which may have contributed to the onset of their ED. It is important to recognise that EDs serve a function which can include coping with past events. Our role as clinicians is to listen to, and support, service users to explore the unique meaning their ED holds for them and why they have consequently come to value it.

*I have listened to stories of social isolation, discrimination, prejudice and bullying. Stories of abuse, neglect, abandonment or inconsistent care. Many have experienced multiple unpredictable life events or grew up in emotionally invalidating households where they learnt that their emotions were unacceptable and need to be squashed down. Others were raised in environments where love, acceptance and approval were conditional on achievement and accomplishment, and their self-worth became dependent on achieving excellence.*

Given these experiences, it is unsurprising that people are motivated to change their eating habits if, for them, it helps instill a sense of control. However, it is crucial we don’t oversimplify the construct of control or view it as an undesirable personality trait. When you explore what control encapsulates for people, it often includes avoiding or numbing intolerable emotions, creating a semblance of stability and predictability in an uncertain or unsafe world, or having a sense of power, agency and influence over their life. Surely things, as humans, we all desire?

Having been invited into this discussion around control, one of our most impactful reflections was that, as professional service providers, we have been surrounded by the control framework—and this largely goes unquestioned. There may be value in taking time to step back and question whether this has implications for the lived experience of individuals with EDs and also how they are perceived by others.

*As a professional, what strikes me is that most of us have been trained to blindly accept the notion of EDs (Anorexia Nervosa especially) as an attempt to control one’s life because, Bruch and her contemporaries told us that was an inherent feature of the disease, and it wasn’t questioned. I think my professional views were shaped, thankfully, more by my own lived experience (for which this view didn’t resonate, and I resented the pejorative implications of being viewed as a ‘control freak’) and by my feminist-oriented early education, as well as my adoption of feminist-oriented perspectives of EDs early in my career.*

Despite some differences in our approaches, we both reflected upon how we inevitably seek to see the bigger picture and try not to fall into the trap of reductionism. That said, we appreciate that the field could do more to critique the specific language that is universally used in relation to control, hence our involvement with this position piece. Reframing EDs through the language we use could potentially be very powerful. For example, reframing in a manner which encapsulates the context, rather than pathologising EDs as a disorder that ‘occurs within’ the individual. Researchers [31] have called for a move away from *“disordering discourse to transformative dialogue”* for mental health conditions; highlighting how disordering discourse can lead to stigmatisation, client blame, feelings of disempowerment and deteriorated relationships between clinician and client. We believe the discourse around control and EDs could potentially be an example of this.

*By re-framing eating disorders as a way to avoid danger and meet core human needs such as safety, care, mastery and connection, we can help to communicate non-judgement and compassion, in the hope that the individual will in time take this stance towards themselves.*

We should seek to avoid service users and service providers potentially placing *overemphasis* on control as the underlying factor of EDs. Excessive emphasis runs the risk of influencing individuals to view their ED solely as a means of control, robbing them of the opportunity to narrate their experience first-hand. In turn, this could prevent clinicians identifying vital information that could have supported the individual’s recovery process. We must do what we can to help our clients identify and voice their own truths in their processes of recovering their life from an ED. Continuing to grow and constructively critique current practices is essential for continued growth and improvement in any field.

## Conclusion and suggestions for the future

This position paper aims to inspire critical thinking of how the language we use can significantly impact how we—both as individuals and as a society—conceptualise, treat and/or experience EDs. Through listening to the voices of lived experience, clinicians, researchers and the wider public, we can move towards the co-construction of more inclusive knowledge and more appropriate and/or helpful framing. In doing so, the person's expertise is privileged, including the meanings they ascribe to the ED experience, rather than being inadvertently dismissed by over-reliance on professional discourses, including a control discourse.

Having highlighted some of the potential issues around current discourse, it is important not to stop there but to identify key suggestions for how we, as a society, can move forward. This includes raising key questions for future research, and how we can encourage one another to think more critically about the language we use and the impacts this can have.

We propose the following steps for moving forward based on the overarching premise that eschews the assumption and unexamined use of a control framing for EDs, and instead moves towards a more critical consideration of the terminology used:

1. *For clinicians and researchers*, this should include critical self-reflection and awareness of the language being used. Where possible, a person-centered approach should be promoted which encourages the adoption of methodologies and clinical approaches which explore *i. How individuals relate to control in relation to their own ED, ii. Where these perspectives originated from, and iii. Whether these framings are beneficial or detrimental (and why).* Individuals living with an ED(s) should be supported to explore their own experiences and to identify the language that best explains their experiences, emotions and behaviours. This could include approaches based on guided self-discovery, e.g., CBT-E [32] and interventions that position the person as the expert of their life, including the language they use to depict their experiences (e.g., Narrative Therapy [33] and Motivational Interviewing [34]).
2. *At a wider societal level*, we should resist oversimplifying psychological disorders, such as EDs. Simple, short words (e.g., control) may facilitate easy, untaxing explanation but this can come at a cost (e.g., negative stigma, reductionism). This should be linked to improved education around EDs across all sectors.
3. *Policy makers* should do more to implement and/or enforce media guidelines around responsible reporting on eating disorders. The UK Eating Disorder Charity BEAT has made good progress by releasing their own media guidelines [35]. The BEAT guidance focuses on dispelling common ED myths and avoiding triggering content. We would like to see more organisations, governmental bodies and policy makers adopting and promoting clear, easy to follow guidance. We recommend that this is developed to include careful consideration of more nuanced language (such as control) and how use of such language can be potentially reductionist, stigmatising, and/or misleading.

In writing this position paper, the authors hope to inspire critical thinking and appropriate change within the dominant ED discourse. ED support and awareness could be improved by moving away from habitual, non-reflective and/or overemphasised references to control—and towards critical consideration around framing, language impact and context.

## Data Availability

Not applicable.
